# Supplementary material for: Interleukin 26 Induces Macrophage IL-9 Expression in Rheumatoid Arthritis
Source: Int J Mol Sci. 2023 Apr 19;24(8):7526. doi: 10.3390/ijms24087526 (PMC10139149; doi:10.3390/ijms24087526)
Supplement: Supplementary file 1 [file ijms-24-07526-s001.zip › ijms-2347151-supplementary.pdf]

International Journal of
Molecular Sciences

MDPI

Supplementary Figure

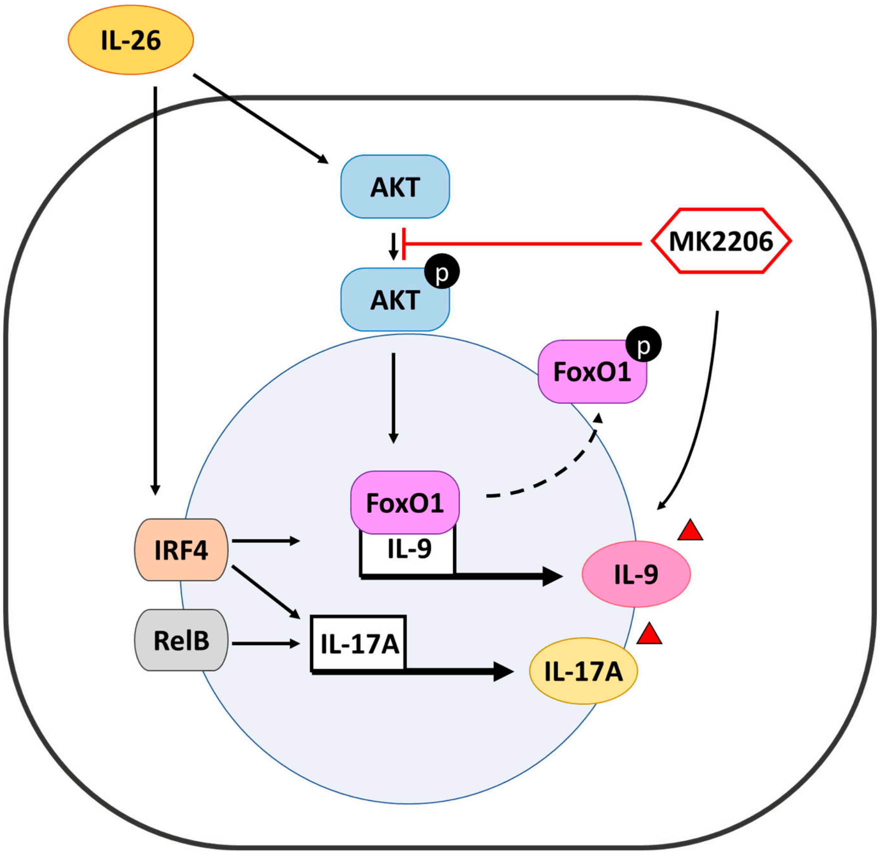

Figure S1. The diagram and mechanisms of IL26 stimulates macrophages. IL-26 stimulation promotes macrophage cells upregulates IL-9 and IL-17A expression by activating IRF4 and AKT-FoxO1pathway. Blockage of activating AKT inclines the phopho-FoxO1 to functional FoxO1 and increases IL-9 expression.

Supplementary Tables

Table S1. Primary antibodies

FITC anti-mouse CD80	104706, Biolegend, San Diego, CA, USA
PE anti-mouse IL-9	514104, Biolegend, San Diego, CA, USA
Alexa Fluor® 647 anti-mouse IL-17A	506912, Biolegend, San Diego, CA, USA
FITC anti-human CD80	305206, Biolegend, San Diego, CA, USA
PerCP/Cyanine5.5 anti-human IL-9	507610, Biolegend, San Diego, CA, USA
PE anti-mouse IL-17A	506904, Biolegend, San Diego, CA, USA
APC anti-human CD14	301808, Biolegend, San Diego, CA, USA
CD68 Monoclonal Antibody, FITC	11-0689-41, Thermo Fisher Scientific, Waltham, MA, USA
Rabbit anti IL9 Antibody	DF2532, Affinity Biosciences, Jiangsu province, China.
Mouse amti IL26/AK155	MAB1375, R&D Systems, Minneapolis, MN USA
Rabbit anti IRF4	#4948, Cell Signal, Danvers, MA, USA

Mouse anti RelB	sc-48366, Santa Cruz, Dallas, TX, USA
Mouse anti PU.1	sc-390659, Santa Cruz, Dallas, TX, USA
Rabbit anti mouse RORγT	ab207082, Abcam, Cambridge, UK
Mouse anti AKT1	680302, Biolegend, San Diego, CA, USA
Rabbit anti p-AKT1 (S473)	ab81283, Abcam, Cambridge, UK
Rabbit anti GAPDH	#5174, Cell Signal, Danvers, MA, USA

Table S2. Secondary antibodies for western blot

Goat Anti-Mouse IgG (H+L)	115-035-003, Jackson ImmunoResearch, West Grove, PA, USA
Goat Anti-Rabbit IgG (H+L)	111-035-003, Jackson ImmunoResearch, West Grove, PA, USA
Alexa Fluor® 594 AffiniPure Donkey Anti-Mouse IgG (H+L)	715-585-150, Jackson ImmunoResearch, West Grove, PA, USA

Table S3. Primer for Real Time-PCR

Target	Sequence
m_IRF4 Forward	CCTCTTCAAGGCTTGGGCAT
m_IRF4 Reverse	TCAGGGGCATAATCCCTCCA
m_RelB Forward	GGTCTCCAGGACCACAGAAAT
m_RelB Reverse	CTGTGATGACCAGGTACGGC
m_PU.1 Forward	AACCAAGTCATCCGATGGAGGG
m_PU.1 Reverse	GCCCTGCAATGTCAAGGGAG
m_RORγT Forward	TACCCTACTGAGGAGGACAGG
m_RORγT Reverse	TTGACAGCATCTCGGGACA
m_GAPDH Forward	ACAGTCTTCTGGGTGGCAGTGAT
m_GAPDH Reverse	GTGAGGCCGGTGCTGAGTATGT
h_IRF4 Forward	GCCAAGATTCCAGGTGACTC
h_IRF4 Reverse	CTGGCTAGCAGAGGTTCTACG
h_RelB Forward	GGAAGTAGACATGAATGTGGTGAG
h_RelB Reverse	GCTCTGATGTGTTTGTGGATTT
h_PU.1 Forward	CCTGAGGGGCTCTGCATTG
h_PU.1 Reverse	GAAGTCCCAGTAATGGTCGCT
h_RORgT Forward	GCACCCCTCACAGGTGATAA
h_RORgT Reverse	GAGAGCTAGGTGCAGAGCTT
h_GAPDH Forward	AGCCACATCGCTCAGACAC
h_GAPDH Reverse	GCCCAATACGACCAAATCC
